# The application of the 150° oblique tangential fluoroscopic view to detect the posterosuperior femoral neck screw in–out–in intraoperatively

**DOI:** 10.1038/s41598-022-17221-z

**Published:** 2022-07-27

**Authors:** Jian Zhang, Xin Tang

**Affiliations:** 1 Dalian Medical University, Dalian, 116044 Liaoning Province China; 2 Department of Orthopedic Trauma, The First Affiliated Hospital of Dalian Medical University, 222 Zhong Shan Road, Xi Gang District, Dalian, 116011 Liaoning Province China

**Keywords:** Trauma, Bone, Skeleton

## Abstract

This study investigates the application of the 150° tangential fluoroscopic projection as a novel fluoroscopic view to detect the posterosuperior screw in–out–in (IOI) in the cannulated screws fixation of femoral neck fractures. A retrospective analysis was conducted including 33 patients with femoral neck fractures enrolled from April to November 2021. All patients underwent closed reduction and internal fixation with cannulated screws under intra-operative C-arm fluoroscopy. The posterosuperior femoral neck screw position (whether in–out–in and the distance to the femoral neck cortex) was evaluated from the standard anteroposterior (AP), lateral view, and tangential view images. Postoperative computed tomography (CT) scan results are considered the gold standard for detecting the femoral neck screw locations. Of 33 patients, no femoral neck screws were found to be placed IOI under the standard AP and lateral views. The tangential view revealed the posterosuperior screw was IOI in 8 patients, whereas the average distance between the posterosuperior screw and the posterior femoral neck cortex was 2.73 ± 1.06 mm under the standard lateral view. Postoperative CT verified that posterosuperior screw was placed IOI in these 8 patients. In the other 25 patients with the tangential view showed the posterosuperior screw completely contained in the femoral neck, the average distance between the posterosuperior screw and the posterior femoral neck cortex was 5.48 ± 1.26 mm under the standard lateral view and 2.76 ± 1.08 mm under the tangential view, with a statistically significant difference between the two groups (p < 0.05). Post-operative CT demonstrated that the femoral neck screws were completely contained in the femoral neck in these 25 patients. Intra-operative tangential view of 150° can effectively identify the posterosuperior screw IOI in the cannulated screws fixation of femoral neck fractures. Based on our study, we highly recommend the tangential view as a routine intraoperative fluoroscopic angle to detect the posterosuperior screw IOI.

## Introduction

Hip fractures cripple 4.5 million individuals worldwide each year, of which femoral neck fractures account for about 53%^1^. Inverted triangular cannulated screws were commonly used in the treatment of femoral neck fractures according to the three point principle^2^. Lindequist et al. demonstrated that optimal support of the femoral neck cortex can be achieved when the distance between the screw and the femoral neck cortex is less than 3 mm^3^.

**Figure 1:**
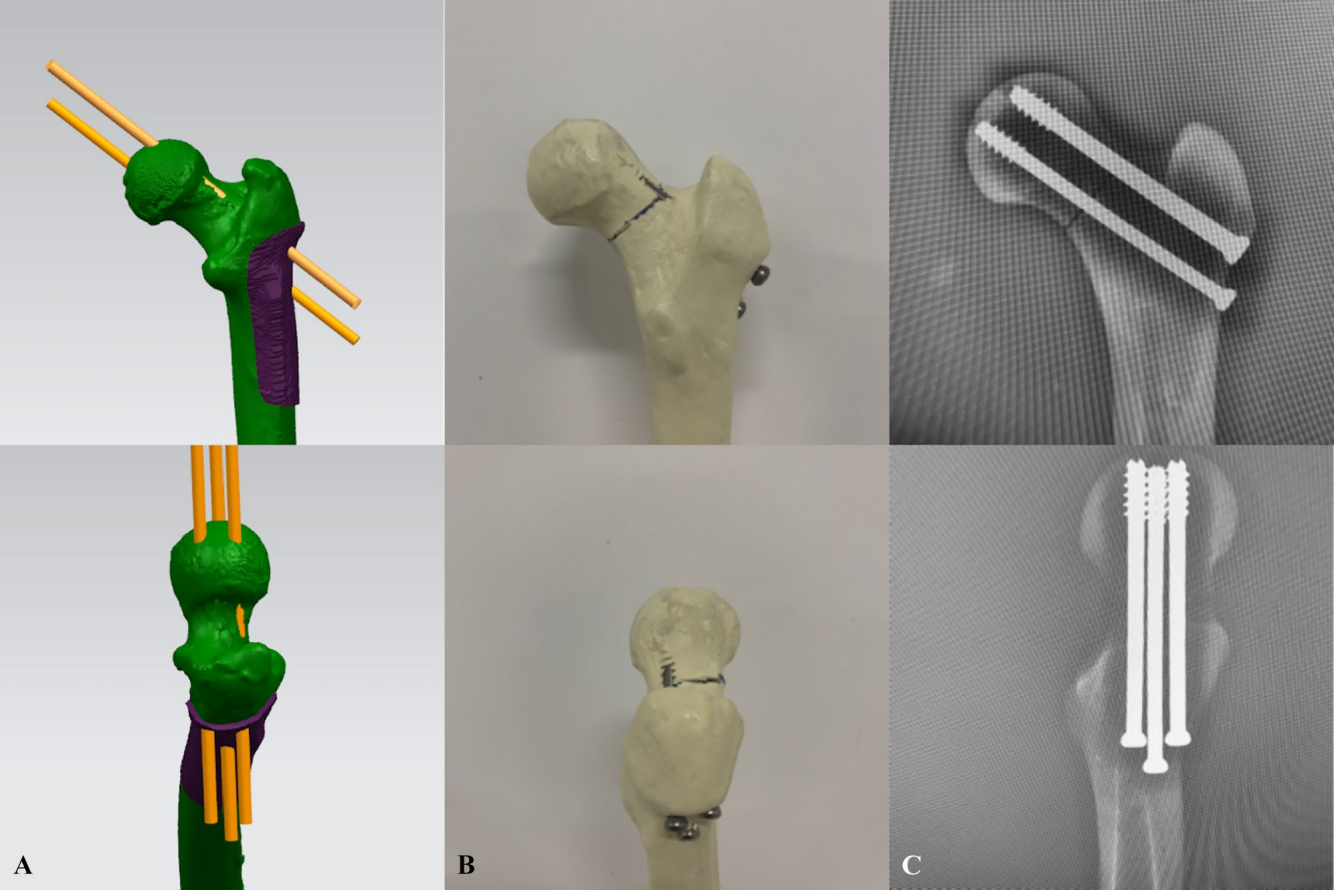
The limitations of the standard AP and lateral views in detecting IOI. (**A**) Software simulation IOI. (**B**) Specimen simulation IOI. (**C**) The standard femoral neck AP and lateral views showed all screws were contained within the femoral neck.

Currently, in clinical practice, the standard femoral neck AP and lateral views are used as intraoperative fluoroscopic evaluation means to assess the quality of fracture reduction and screw placement^4^. However, previous literatures revealed that the standard femoral neck AP and lateral views couldn’t accurately reveal the IOI posterosuperior femoral neck screw^5,6^. For further validation, we simulated the posterosuperior screw IOI in the inverted triangle cannulated screws fixation of femoral neck fractures via Unigraphics NX 12.0 (Siemens PLM Software), while all screws are contained within the femoral neck under the standard AP and lateral views. Subsequently, we utilized an artificial femur model simulating inverted triangular cannulated screws internal fixation of femoral neck fractures to determine the limitations of the standard AP and lateral views in detecting IOI (Fig. 1).

**Figure 2:**
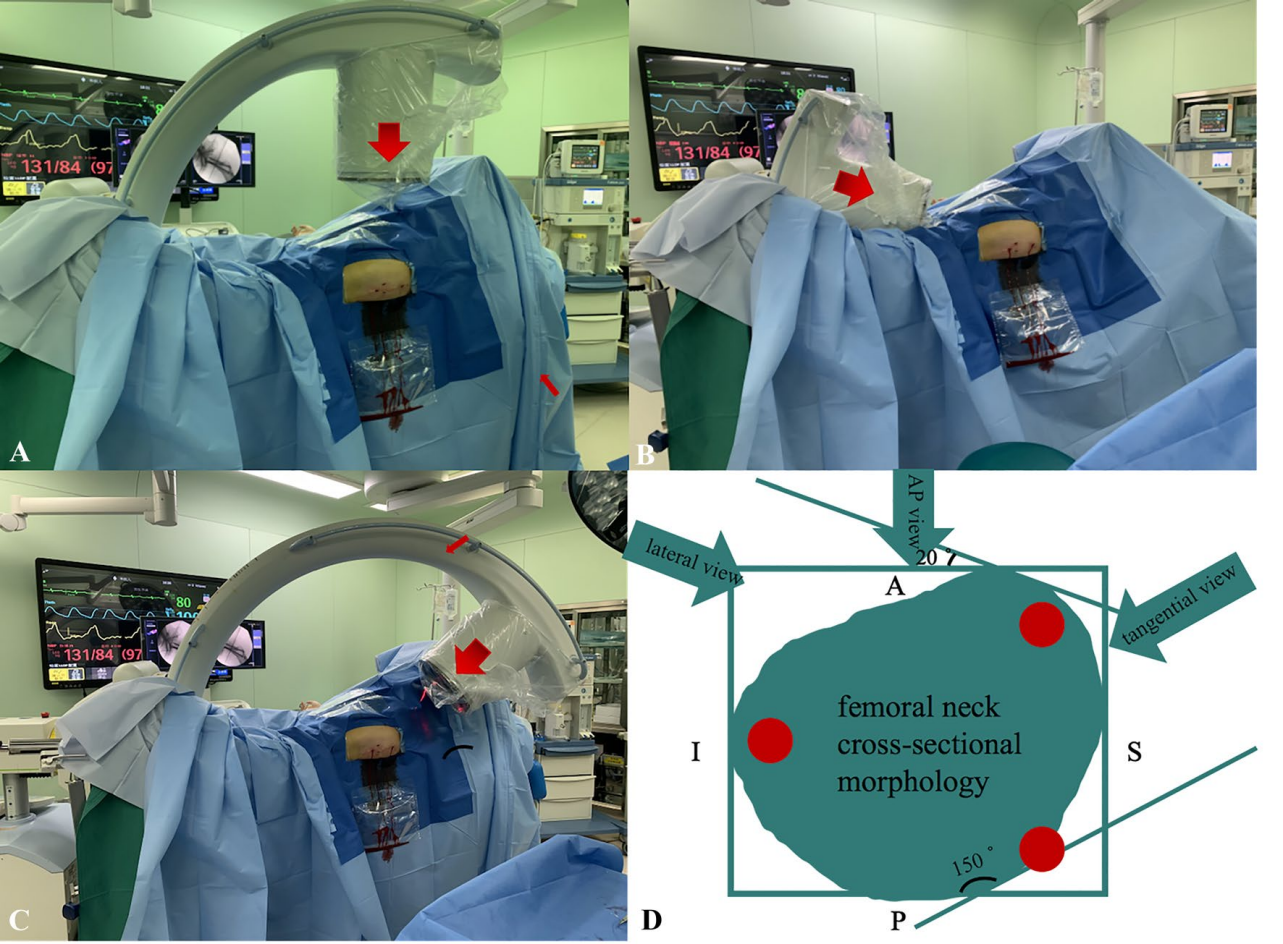
(**A**) Positioning of C-arm during the standard femoral neck AP view, (**B**) positioning of C-arm during the standard femoral neck lateral view, (**C**) positioning of C-arm during the 150° tangential views, (**D**) the red circles represent femoral neck screws, the green oblique oval represents the femoral neck cross-sectional.

Anatomical studies have shown that the cross-sectional morphology of the femoral neck is approximately obliquely elliptical, with more defects in the posterosuperior and anteroinferior areas^7,8^. With increasing osteoporosis, the trabecular comes to lie progressively more antero-superiorly within the femoral neck^9^. Precisely for this reason, the posterosuperior screw has a high cortical breakage rate, creating an In–Out–In (IOI) screw. Based on the anatomy of the proximal femur, we proposed a hypothesis to identify the presence of posterosuperior screw IOI by fluoroscopy of the tangential position of the posterosuperior femoral neck cortex, usually at 150 °C-arm rotations (Fig. 2). Our study aimed to introduce a new fluoroscopic view called the tangential view, which can be used to detect the posterosuperior screw in–out–in and to signify its value in clinical applications.

## Materials and methods

### Patients

The study retrospectively enrolled patients with femoral neck fractures who underwent closed reduction and internal fixation with cannulated screws under two-dimensional (2D) C-arm fluoroscopy in our level-1 trauma center from April to November 2021. Inclusion criteria were: the time interval from injury to operation < 3 weeks, Age > 18 years, intraoperative C-arm fluoroscopic (including the standard femoral neck AP, lateral and tangential views) and post-operative CT (both 2D and 3D imaging) of the femoral neck were available. Exclusion criteria included: pathological femoral neck fractures, patients with anatomical deformities of the proximal femur, patients with multiple fractures of the ipsilateral femur, patients with previous hip fractures. The study population consisted of 33 patients with femoral neck fractures (10 men and 23 women; 15 left and 18 right) and a mean age of 64.38 ± 12.62 years (range 43–86 years). Hip X-ray and CT were completed before surgery. AO Foundation and Orthopaedic Trauma Association (AO/OTA) classification revealed 7 cases of type 31B1.1, 9 cases of type 31B1.2, 6 cases of type 31B1.3, 7 cases of type 31B2.2, and 4 cases of type 31B2.3. The institutional review board approved the study and written informed consent was obtained from all patients. This retrospective study was approved by the Ethics Committee of the First Affiliated Hospital of Dalian Medical University (number PJ-KS-KY-2021-248) and performed in accordance with the Helsinki Declaration.

### Treatment protocol

All patients with femoral neck fractures scheduled for surgery in our level-1 trauma center were evaluated preoperatively by an anesthesiologist and medical consultation if necessary. After medical contraindications were eliminated, the surgery was performed immediately by a senior trauma orthopedic surgeon under the direct supervision of the chief of trauma orthopedics. After general anesthesia, closed reduction was performed on the fracture traction table. The C-arm was routinely placed in the crotch between the two legs to observe the fracture reduction quality and the position of the implant. If the quality of the closed reduction is unsatisfactory, use the joystick reduction technique to achieve anatomical position^10^. Fractures were fixed using 7.5 mm diameter semi-threaded, cannulated screws. Inverted triangular cannulated screws were placed according to the three-point principle. Both ends of the screw are fixed in solid bone, respectively the proximal lateral femoral cortex and the subchondral bone of the femoral head. The shafts of screws were positioned against the inner surface of the femoral neck cortex. The surgeon decided whether to place an off-axis screw perpendicular to the fracture line through the greater trochanter to counteract shear forces, depending on the degree of fracture stability. The whole process of internal fixation implantation was under the standard femoral neck AP and lateral views.

After the implantation procedure of the femoral neck with cannulated screws, a comprehensive assessment of the position of the posterosuperior screw involved the observation of radiographs in the standard femoral neck AP, lateral and tangential views. The AP view is the position where the best outline of the greater trochanter can be seen^5^. In the standard lateral view (true sagittal) of the femoral neck, the angle of X-ray provided by the C-arm bulb tube and the ground is approximately 20°, thus eliminating the influence of the anteversion angle of the femoral neck and aligning the femoral head, femoral neck axis, and femoral shaft axis in a straight line^11^. In the tangential view of the femoral neck, the angle of X-ray provided by the C-arm bulb tube and the ground is approximately 150° so the projection plane was tangential to the posterosuperior femoral neck cortex. Under the standard femoral neck AP, lateral and tangential views, observe whether the posterosuperior cannulated screw is in–out–in. If not, measure the distance between the posterosuperior screw and the posterior femoral neck cortex under the standard lateral and tangential views.

### Postoperative imaging evaluation

Plain radiographs and computed tomography scans of the injured hip were performed immediately after surgery. The director of trauma orthopedics reviewed all images to observe whether the posterosuperior screw was IOI. The postoperative computed tomography (both 2D and 3D imaging) results are considered the gold standard because it provided a full range of views of fracture reduction and cannulated screw position^12^. The postoperative CT images were performed with 16-detector spiral CT scanner (GE Health care, LightSpeed CT, Waukesha, WI), and the scanning parameters were as follows: layer thickness, 0.625 mm; tube voltage, 120 kVp; pitch, 1.375; matrix, 512 × 512.

### Statistical analysis

Descriptive variables are presented in the text as means and standard deviations. The evaluation of the CT scan is the reference to assess the position of the posterosuperior cannulated screw. Student's T-Test was used to compare the distance between the posterosuperior screw and the posterior femoral neck cortex under the standard lateral and tangential views. Statistical significance was defined as p < 0.05.

### Ethics approval and consent to participate

The collected data was anonymized and de-identified before data analysis. The Institutional Review Board of First Affiliated Hospital of Dalian Medical University granted a waiver of written informed consent and provided authorization for this study (number PJ-KS-KY-2021-248).

## Results

**Figure 3:**
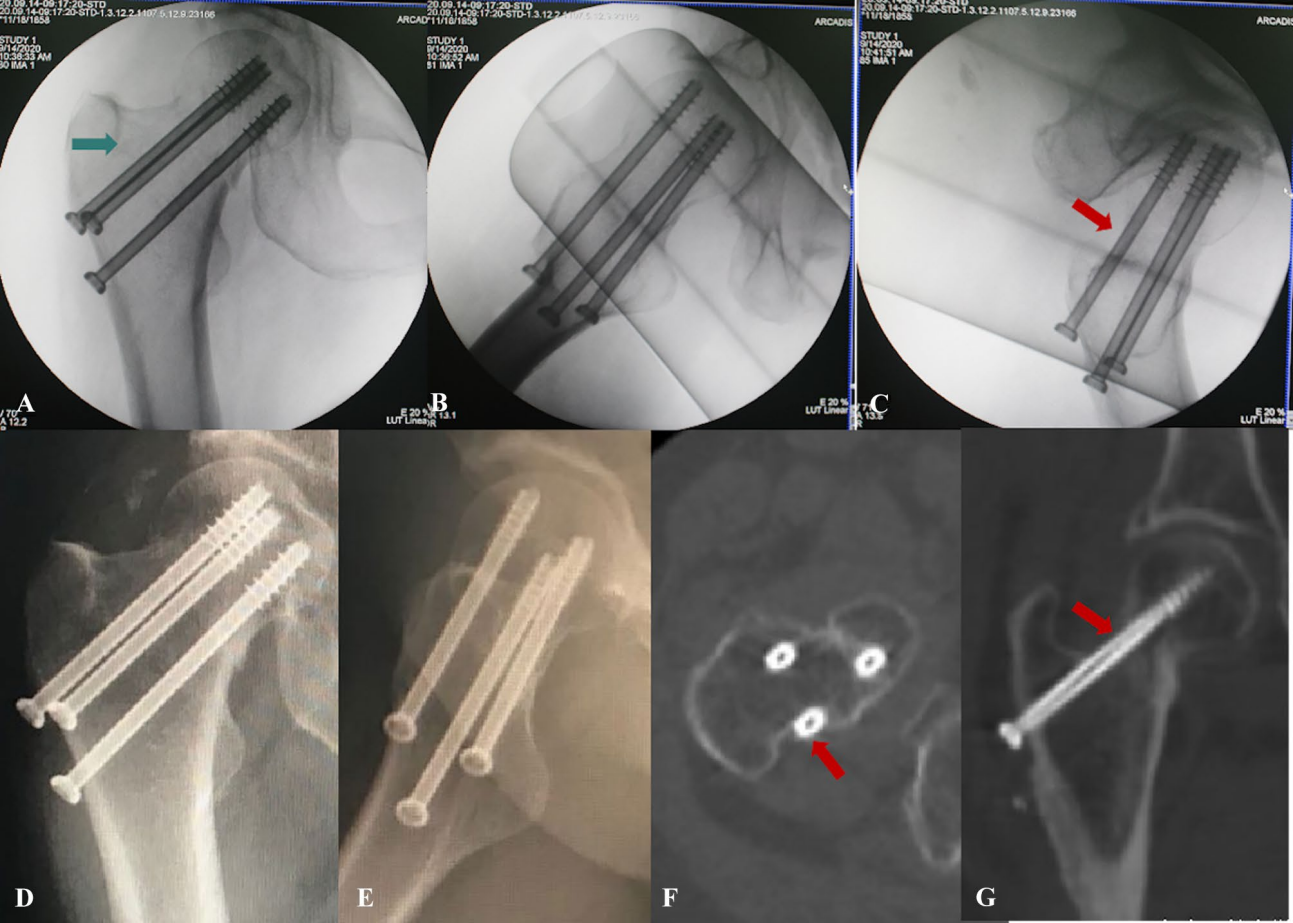
The posterosuperior femoral neck screw placed caudal to the inferior margin of the piriformis fossa in–out–in. The green arrow represents the piriformis fossa radiographic landmark. The red arrow represents the posterosuperior femoral neck screw IOI. (**A**,**B**) Intra-operative the AP and lateral views showed all screws were contained within the femoral neck. (**C**) Intra-operative the tangential view showed the posterosuperior screw IOI. (**D**,**E**) Postoperative plain radiographs showed all screws were contained within the femoral neck. (**F**,**G**) Postoperative CT showed the posterosuperior femoral neck screw IOI.

There were 10 males and 23 females with a mean age of 64.38 ± 12.62 years (range 43–86 years). All femoral neck fractures were satisfactorily reduced, almost to the anatomical position. Of 33 patients, no femoral neck screws were found to be placed IOI under the standard AP and lateral views. The tangential view revealed the posterosuperior screw was IOI in eight patients, whereas the average distance between the posterosuperior screw and the posterior femoral neck cortex was 2.73 ± 1.06 mm under the standard lateral view. Postoperative CT (both 2D and 3D imaging) through a fine (2 mm) cut of the femoral neck revealed that the posterosuperior screw was in–out–in in 8 patients, the same as under the tangential view (Fig. 3). In the other 25 patients with the tangential view showed the posterosuperior screw completely contained in the femoral neck, the average distance between the posterosuperior screw and the posterior femoral neck cortex was 5.48 ± 1.26 mm under the standard lateral view and 2.76 ± 1.08 mm under the tangential view, with a statistically significant difference between the two groups (p < 0.05). Post-operative CT demonstrated that the femoral neck screws were completely contained in the femoral neck in these 25 patients (Table 1).

**Table 1:** Evaluation of the posterosuperior femoral neck screw position based on intra-operative fluoroscopy and post-operative CT.

Post-op 3D CT	Intra-op fluoroscopy		No. of cases
	Lateral view	Tangential view	
In–out–in	Contained (d = 2.73 ± 1.06)	In–out–in	8
Contained	Contained (d = 5.48 ± 1.26)	Contained (d = 2.76 ± 1.08)	25

## Discussion

**Figure 4:**
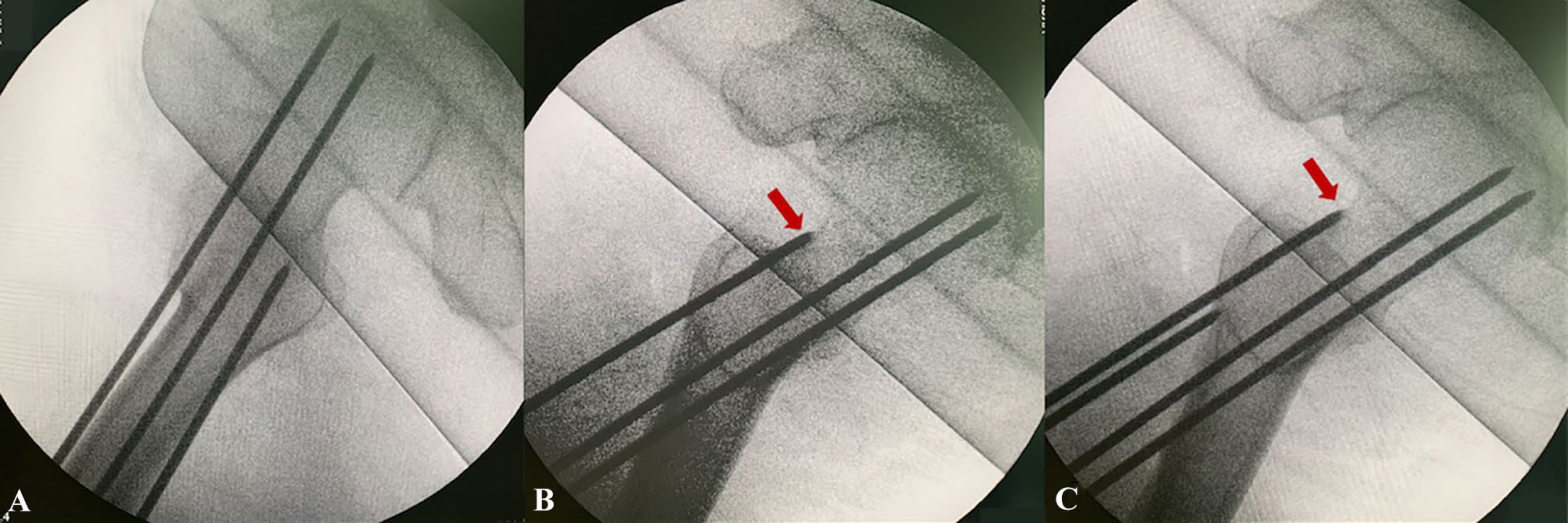
Tangential view instructs femoral neck screw guide wire implantation. (**A**) Intra-operative the lateral views showed the posterosuperior screw guide wire were contained within the femoral neck. (**B**) Intra-operative the tangential view showed the posterosuperior screw guide wire IOI. (**C**) Adjust the guidewire under the tangential view.

The purpose of our study was to investigate the value of the intra-operative tangential view to determine the posterosuperior femoral neck screw IOI in clinical applications. This study observed that the tangential view was more sensitive than the standard lateral view in distinguishing the posterosuperior screw IOI. Therefore, we recommend that the tangential view should be used as a routine C-arm machine fluoroscopic plane during the implantation of the cannulated screw guide wire for internal fixation of femoral neck fractures. When the tangential view reveals that the femoral neck screw guide wire is IOI or very close to the femoral neck cortex, the position of the guidewire should be promptly adjusted (Fig. 4).

Bony violation during screws fixation has been widely reported in the orthopaedic literature. Routt et al. suggested iliosacral screw insertion into the sacral isthmus region required avoidance of IOI to reduce the risk of damage to the adjacent neurovascular structures^13^. Du et al. recommended the C2 pedicle screw IOI to provide multicortical 3-column rigid fixation in the patients with basilar invagination and atlantoaxial dislocation^14^. Ramesh et al. proposed anterior column lag screw IOI in the fixation of acetabular fractures to provide rigid stability and minimize the surgical duration, radiation exposure, and intra-operative complications^15^. A previous clinical study demonstrated the posterosuperior screws IOI incidence up to 54% in the inverted triangle cannulated screws fixation of femoral neck fractures. However, the sensitivity of the standard lateral view to identifying the posterosuperior screw IOI was only 39%^6^. In a cadaveric study, 2 orthopaedic traumatologists and 1 musculoskeletal radiologist determined that no screws radiographically breached the posterior and cranial cortex in 10 cadaver specimens under the standard femoral neck AP and lateral views. After dissection, 70% of the specimens emerged with the posterosuperior screw IOI^16^. Aibinder et al. proposed the use of the sequential fluoroscopic rollover images to detect an IOI position after placement of the posterosuperior guide wire into the femoral neck^17^. However, this technique significantly increases the frequency of intraoperative fluoroscopy, leading to surgeons-possible damage by occupational ionizing radiation exposure^18,19^. Therefore, this technique is not recommended for clinical applications. Adams et al. proposed placing the posterosuperior screw to the piriformis fossa inferior margin on AP view to avoid cortical breach during percutaneous screw fixation of femoral neck fractures^5^. However, we considered the screw location was a three-dimensional position so relying on the anatomical signatures on AP view alone was not credible, and our clinical cases validated the inaccuracy of this methodology (Fig. 3). In summary, no research was available to instruct guide wire implantation intraoperatively and thus avoid the femoral neck screw IOI.

However, the influence of IOI screws in the fixation of femoral neck fractures has not been clearly demonstrated. Femoral neck fractures frequently involve complications such as non-union and avascular necrosis, which are associated with disruption of the blood supply to the femoral head^20^. Conventionally, the superior retinacular artery (SRA) derived from the medial femoral circumflex artery is considered to be the main blood supply to the femoral head^21,22^. The SRA ran through the lateral retinaculum which had the form of a quadrilateral plate adjacent to the posterosuperior surface of the femoral neck^23^. The posterosuperior screw was “in–out–in” near the area where the superior retinacular artery enters the femoral neck, which means that there is a high risk of screw perforation invading the artery^16^. Due to the effects of the iatrogenic injury on the intraosseous vascular system, the blood supply of the femoral head is severely deteriorated, leading to non-union and secondary femoral head avascular necrosis^24,25^. Yuan demonstrated that the incidence of avascular necrosis and revision surgery in hips with and without IOI screws was 6% and 6%, respectively; however, due to the width of the confidence intervals, a true clinical difference could not be excluded^6^. We believe this consequence was also related to the small sample volume, and a long-term, large sample follow-up study on the relation between necrosis and iatrogenic injury will be necessary for the future.

**Figure 5:**
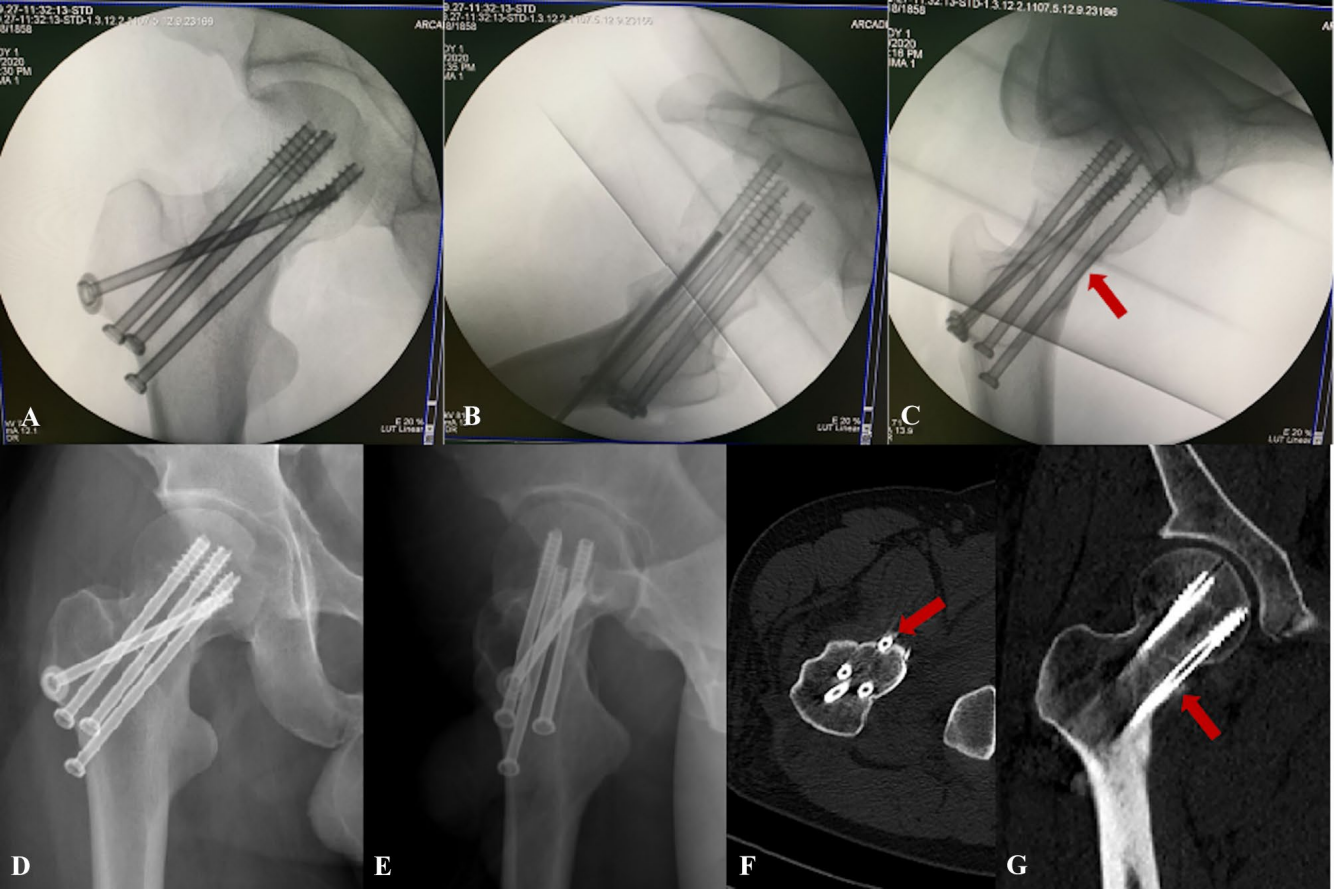
(**A**,**B**) Intra-operative the AP and lateral views showed all screws were contained. (**C**) Intra-operative the tangential view showed the inferior screw IOI. (**D**,**E**) Postoperative plain radiographs showed all screws were contained. (**F**,**G**) Postoperative CT showed the inferior screw IOI. The red arrow represents the inferior screw IOI.

The cross-sectional morphology of the femoral neck is displayed as a rotating forward ellipse^26^. With increasing osteoporosis, the trabeculae come to lie progressively more anterosuperior within the femoral neck, with a larger posterosuperior defect^9^. Therefore, the posterosuperior region is a risk area for bony violation during screw fixation of femoral neck fractures. This also explains that the screws appearing well contained in the standard view might have actually perforated the posterosuperior neck. It was worth mentioning that we found one patient with the inferior screw that was IOI in the postoperative CT (Fig. 5). Intraoperative standard AP and lateral fluoroscopy showed that the inferior screw was completely contained in the femoral neck, while the tangential view showed the inferior screw was IOI. This might be attributed to the inferior screw being implanted anteriorly and penetrated anteroinferior through the femoral neck, while the tangential fluoroscopy tangential to both the posterosuperior and anteroinferior cortices could reveal the accurate screw position.

In our clinical practice, we also concluded certain experiences to avoid IOI. Apply tangential view as a conventional fluoroscopic angle to adjust the guidewire. We usually implant the posterosuperior screw lower than the anterosuperior screw to avoid bony violation, thus creating an oblique triangle configuration^27^. Emphasize the importance of intraoperative manipulation of the senses. A sudden loss of resistance during the cannulated screw guidewire insertion, followed by a sudden reappearance of resistance, may suggest the guidewire IOI. When the femoral neck is narrow, such as in elderly women, we will replace the posterosuperior crew with a 6.5 mm diameter screw to guarantee mechanical strength while reducing the risk of the posterosuperior femoral neck screw IOI.

However, there were some limitations to this study. This was a retrospective study and the number of cases was minor, which may be responsible for the high sensitivity of the tangential view to detect the posterosuperior femoral neck screw IOI. Therefore, a larger multicenter prospective study is necessary in the future to evaluate the sensitivity and sensitivity of the tangential view to detect the posterosuperior femoral neck screw IOI. Due to the different anatomy of the population, 150° tangential fluoroscopy may not apply to all patients. A large sample of femoral neck cross-sectional anatomical studies is necessary to obtain a more accurate tangential position projection angle. Intraoperative anatomical reduction is paramount, and non-anatomical reduction can also lead to the inaccuracy of the tangential view. This was only a radiographic comparison study, and we hope to conduct a larger multicenter study subsequently to investigate the effect of IOI screws on the clinical prognosis and complications of femoral neck fractures.

## Conclusion

Intra-operative tangential view of 150° can effectively identify the posterosuperior screw IOI in the cannulated screws fixation of femoral neck fractures. Based on our study, we highly recommend the tangential view as a routine intraoperative fluoroscopic angle to detect the posterosuperior screw IOI.

## Data Availability

All data generated or analyzed during this study are included in this published article.

## Abbreviations

AP: Anteroposterior
IOI: In–out–in
CT: Computed tomography
2D: Two dimensions
3D: Three dimensions
AO/OTA: AO foundation and orthopaedic trauma association
SRA: Superior retinacular artery
